# Prenatal inflammation enhances antenatal corticosteroid–induced fetal lung maturation

**DOI:** 10.1172/jci.insight.139452

**Published:** 2020-12-17

**Authors:** Augusto F. Schmidt, Paranthaman S. Kannan, James Bridges, Pietro Presicce, Courtney M. Jackson, Lisa A. Miller, Suhas G. Kallapur, Claire A. Chougnet, Alan H. Jobe

**Affiliations:** 1 Division of Neonatology and Pulmonary Biology, Cincinnati Children’s Hospital Medical Center, Cincinnati, Ohio, USA.; 2 Department of Pediatrics, University of Cincinnati, Cincinnati, Ohio, USA.; 3 Division of Immunobiology, Cincinnati Children’s Hospital Medical Center, Cincinnati, Ohio, USA.; 4 Department of Anatomy, Physiology and Cell Biology, School of Veterinary Medicine, UCD, Davis, California, USA.

**Keywords:** Development, Pulmonology, Apoptosis, Expression profiling, Extracellular matrix

## Abstract

Respiratory complications are the major cause of morbidity and mortality among preterm infants, which is partially prevented by the administration of antenatal corticosteroids (ACS). Most very preterm infants are exposed to chorioamnionitis, but short- and long-term effects of ACS treatment in this setting are not well defined. In low-resource settings, ACS increased neonatal mortality by perhaps increasing infection. We report that treatment with low-dose ACS in the setting of inflammation induced by intraamniotic lipopolysaccharide (LPS) in rhesus macaques improves lung compliance and increases surfactant production relative to either exposure alone. RNA sequencing shows that these changes are mediated by suppression of proliferation and induction of mesenchymal cellular death via TP53. The combined exposure results in a mature-like transcriptomic profile with inhibition of extracellular matrix development by suppression of collagen genes *COL1A1*, *COL1A2*, and *COL3A1* and regulators of lung development *FGF9* and *FGF10*. ACS and inflammation also suppressed signature genes associated with proliferative mesenchymal progenitors similar to the term gestation lung. Treatment with ACS in the setting of inflammation may result in early respiratory advantage to preterm infants, but this advantage may come at a risk of abnormal extracellular matrix development, which may be associated with increased risk of chronic lung disease.

## Graphical abstract

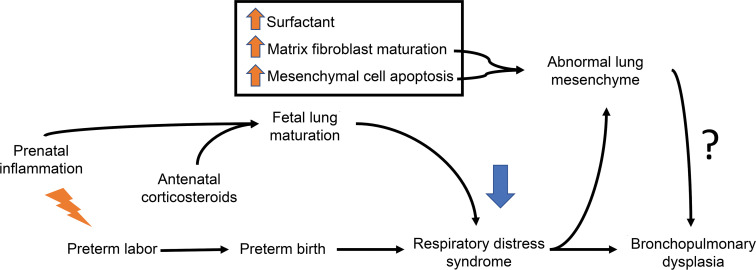

## Introduction

Respiratory complications are a frequent cause of morbidity and mortality among extreme preterm infants (1). Lung adaptation to air breathing requires complex interactions of diverse cell types that will allow the transition to extrauterine life (2). Some of these processes can be accelerated by administration of antenatal corticosteroids (ACS). ACS causes structural and biochemical lung maturation and decreases the rate of respiratory distress syndrome and mortality of preterm newborns in high–medical resource settings (3, 4). Decreased incidence of respiratory distress syndrome (RDS) has also been associated with chorioamnionitis and prenatal inflammation (5, 6). Although this association in clinical studies has been inconsistent due to the variability of exposures and in clinical diagnoses (7), it is congruent with the observation of improved lung compliance and increased surfactant production in animal models of chorioamnionitis (8, 9).

The majority of preterm infants are exposed to inflammation prior to preterm birth with or without identification of an infectious age (10). This process often involves chronic, low-grade inflammation that can only be detected upon histologic examination of the placenta or analysis of the amniotic fluid (11, 12). With the widespread use of ACS in high-resource settings, most preterm fetuses are exposed to these drugs in the setting of prenatal inflammation. Despite their divergent effects on inflammatory pathways, the coexposure of the fetus to inflammation and ACS results in augmented fetal lung maturation compared with either exposure alone in animal models (13). The interaction of prenatal inflammation and ACS is of particular interest, since it may confer an early respiratory benefit to preterm infants.

Despite the success of ACS in reducing short-term respiratory morbidity and mortality among preterm infants in high-resource settings, in low-resource settings, ACS caused increased neonatal mortality, associated with an increased rate of infection (14), highlighting the complicated interaction between these exposures. Moreover, long-term respiratory complications, especially bronchopulmonary dysplasia (BPD) continues to be the most common morbidity associated with prematurity, despite widespread use of ACS (1, 4). Better understanding of the developmental pathways modulated by prenatal inflammation and ACS and their interaction could open avenues for novel strategies to induce fetal lung maturation that prevent not only short-term, but also avoid long-term respiratory complications. Our goal was to determine the effect of prenatal inflammation on fetal lung maturation and its interaction with ACS on pathways of lung development in preterm rhesus macaque fetuses.

## Results

### Prenatal inflammation induces fetal lung maturation in nonhuman primates.

**Figure 1:**
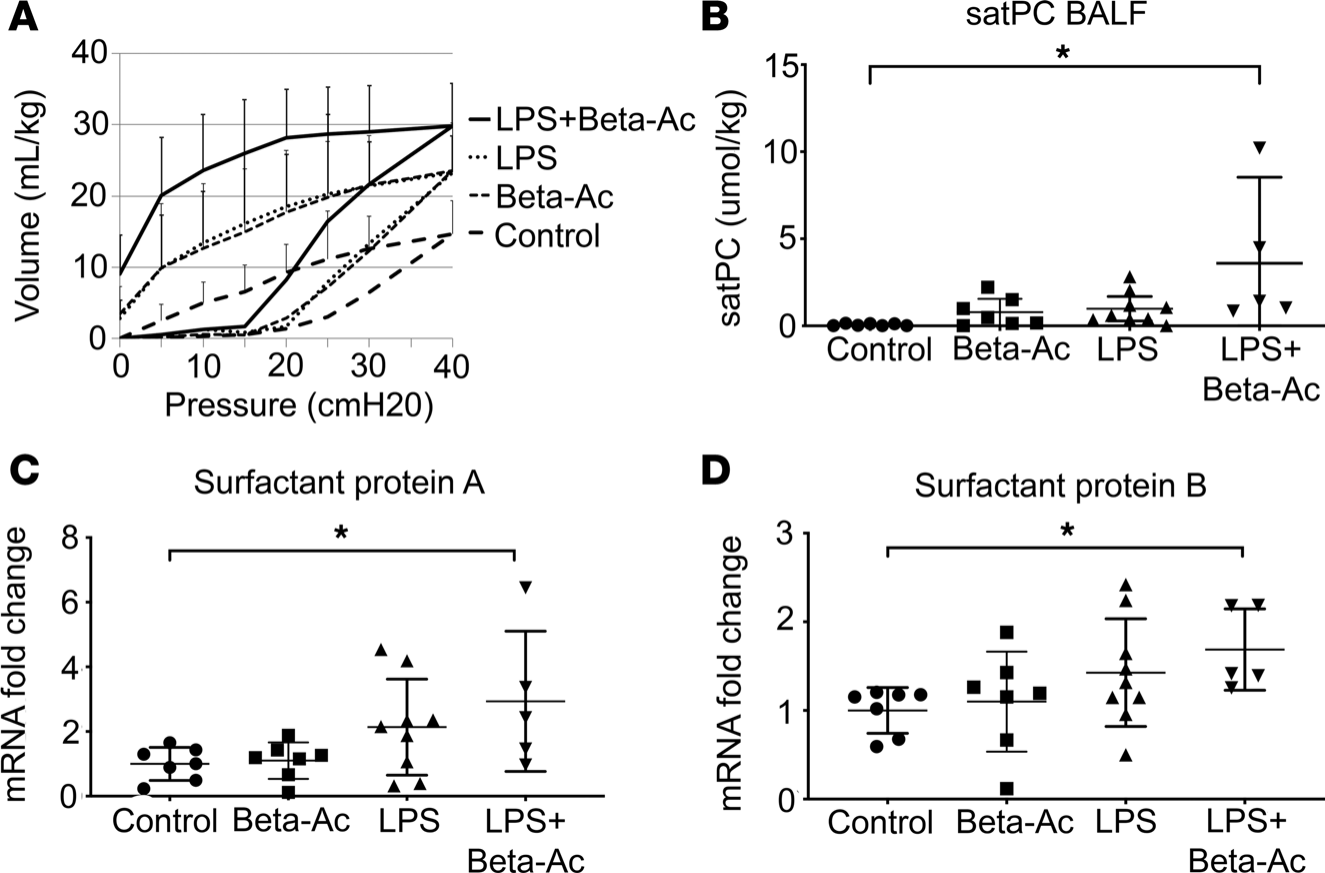
Intraamniotic LPS induces fetal lung maturation and has additive effects to antenatal corticosteroids. (**A**) Pressure volume curves of preterm rhesus macaques exposed to Beta-Ac, intraamniotic LPS, or intraamniotic LPS + Beta-Ac and delivered after 5 days compared with preterm controls and term controls. Intraamniotic LPS improved static lung compliance measured by pressure volume curves to levels similar to Beta-Ac alone, which was further increased by the combined treatment with Beta-Ac. The Beta-Ac–alone curve has been previously published (17). (**B**) Concentration of saturated phosphatidylcholine (satPC) in the bronchoalveolar lavage fluid (BALF) slightly increased after exposure to Beta-Ac or intraamniotic LPS alone and was further increased by the combined treatment with Beta-Ac. (**C**) Real-time PCR for surfactant protein A. (**D**) Real-time PCR for surfactant protein B showed induction of these genes 5 days after combined treatments with intraamniotic LPS and Beta-Ac. Beta-Ac alone and LPS alone did not increase mRNA levels for these proteins after 5 days. *P < 0.05 by ANOVA with Dunnett’s post hoc test for multiple comparisons. Data in pressure volume curve (**A**) presented as mean ± SD; other data (**B**–**D**) presented as mean ± 95% CI with individual data points shown. *n* = 7 controls, 7 Beta-Ac, 9 LPS, and 5 LPS + Beta-Ac.

**Figure 2:**
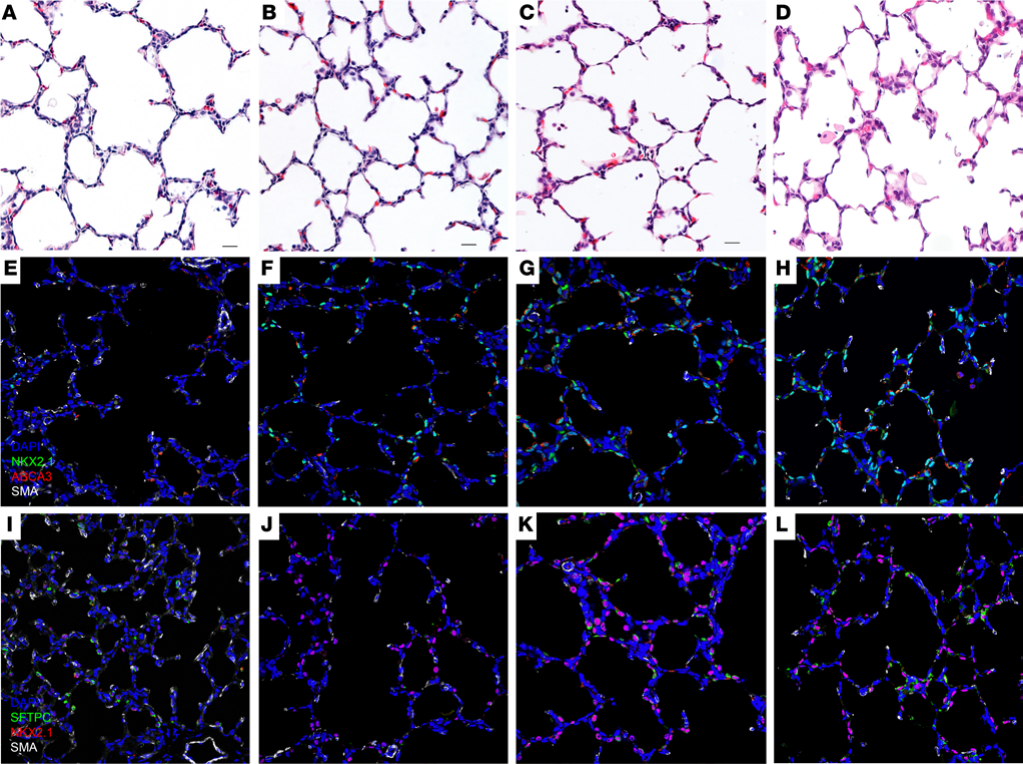
Treatment with Beta-Ac, intraamniotic LPS, and LPS + Beta-Ac induce mesenchymal thinning and increased cellular expression of alveolar epithelial type 2 cell markers. Photomicrographs (original magnification, 20×) of H&E-stained and immunofluorescence for NKX2.1, ABCA3, and SFTPC in lung sections of preterm controls (**A**, **E**, and **I**) and 5 days after treatment with Beta-Ac (**B**, **F**, and **J**), intraamniotic LPS (**C**, **G**, and **K**), and intraamniotic LPS + Beta-Ac (**D**, **H**, and **L**), showing mesenchymal thinning and increased number of cells expressing NKX2.1, ABCA3, and SFTPC after either exposure alone or combined relative to preterm controls. *n* = 7 controls, 7 Beta-Ac, 9 LPS, and 4 LPS + Beta-Ac.

Most preterm infants are exposed to inflammation prior to preterm birth (10), but the effects of this exposure to the neonatal lung function are not well understood. To determine the effects of prenatal inflammation on fetal lung maturation, we administered 1 mg of bacterial lipopolysaccharide (LPS) (*E*. *coli* O55:B5) intraamniotically at 127 days of gestation (term is 165 days). This model has been shown to induce chorioamnionitis and fetal inflammation similar to the clinical scenario (15, 16). To determine the effects of the interaction between inflammation and low-dose ACS, we administered intraamniotic LPS at 1 mg and intramuscular betamethasone-acetate (Beta-Ac) at 0.125 mg/kg to pregnant rhesus macaques at 127 days of gestation (Supplemental Table 1; supplemental material available online with this article; https://doi.org/10.1172/jci.insight.139452DS1). This dose is 37% of the clinical dose of 24 mg of betamethasone and has been shown to induce fetal lung maturation in preterm rhesus macaques (17). Fetuses were delivered by cesarean section 5 days later at 132 days of gestation (80% of term) when the fetal lung is in the saccular stage of development, similar to a preterm human fetus between 28 and 36 weeks’ gestation (18). Exposure to intraamniotic LPS significantly improved fetal lung compliance measured by pressure volume curves and increased the lung volume at a pressure of 40 cmH_2_O (V40) from 15 mL/kg in control animals to 22 mL/kg in LPS-exposed animals. Treatment with Beta-Ac in addition to LPS further increased the fetal lung compliance to a V40 of 40 mL/kg. This value is higher than the average V40 for term animals, which was 27 mL/kg, although statistical analysis is limited, with only 2 animals in the term group (Figure 1A). In similar–gestational age animals, we have shown that treatment with Beta-Ac alone increased the lung compliance and V40 to about 21 mL/kg, similar to the presently observed effect of intraamniotic LPS (17). Of note, the combined exposure to LPS + Beta-Ac resulted in a better deflation stability of the lungs, which indicates higher surfactant concentration in these animals compared with either exposure alone. Combined treatment with LPS and Beta-Ac also resulted in a further increase in the concentration of saturated phosphatidylcholine (satPC), the main component of surfactant, in the bronchoalveolar lavage fluid (BALF), as well as in the lung mRNA of surfactant proteins A and B compared with control, while Beta-Ac or LPS alone did not (Figure 1, B–D). Histological analysis of the lung sections show mesenchymal thinning after either exposure (but it is more striking after exposure to LPS with or without Beta-Ac) and presence of inflammatory cells in the alveolar space after exposure to LPS and LPS + Beta-Ac (Figure 2, A–D). Immunofluorescence and confocal imaging shows an increased number of cells expressing the alveolar epithelial cell markers NKX2.1, ABCA3, and SFTPC after exposure to Beta-Ac, intraamniotic LPS, or LPS + Beta-Ac (Figure 2, E–L).

### Treatment with Beta-Ac in combination with intraamniotic LPS does not suppress fetal or maternal inflammation after 5 days.

**Figure 3:**
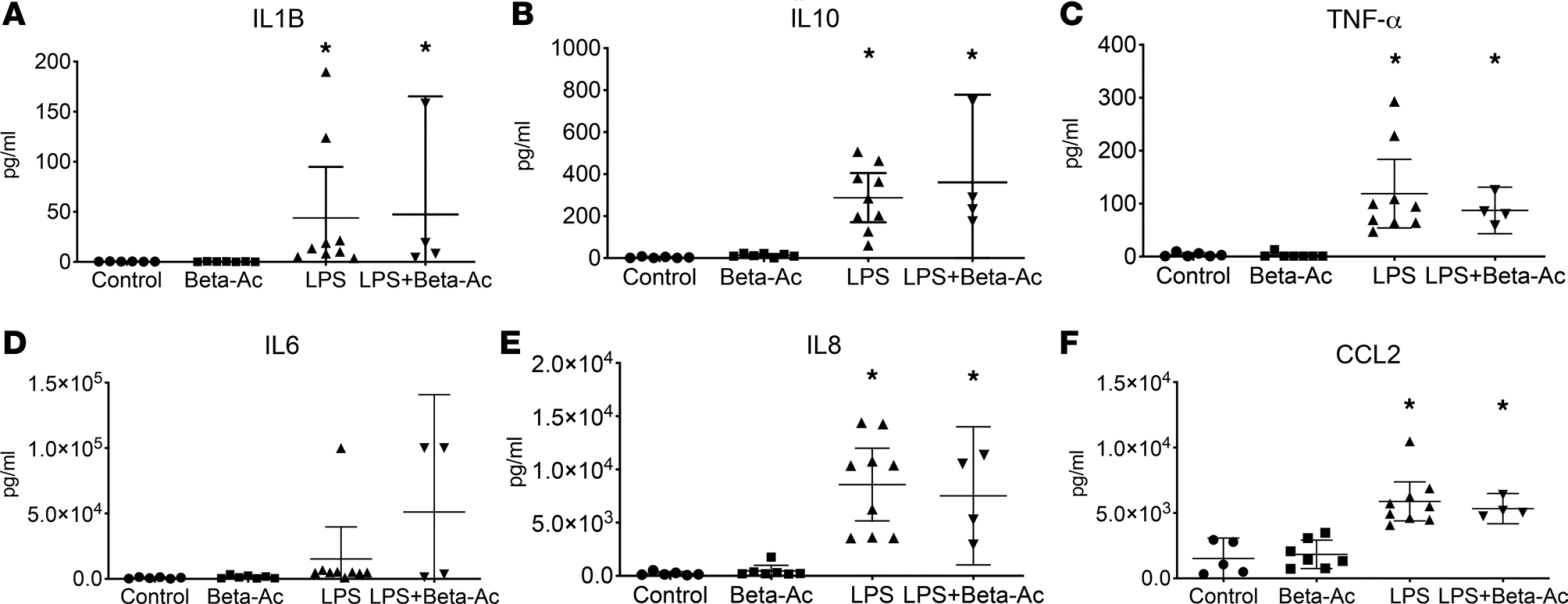
Combined intraamniotic LPS and Beta-Ac treatments given at the same time do not suppress cytokine-mediated inflammation of the amniotic cavity at 5 days. (**A**–**F**) Concentrations of IL-1B (**A**), IL-10 (**B**), TNF-α (**C**), IL-6 (**D**), IL-8 (**E**), and CCL2 (**F**) were measured by ELISA in the amniotic fluid. Beta-Ac alone had no effect on cytokine expression relative to control. Intraamniotic LPS increased the concentrations of all cytokines measured in the amniotic fluid, but treatment with LPS + Beta-Ac did not reduce their concentrations at 5 days relative to LPS. Data presented as mean ± 95% CI with individual data points. *P < 0.05 by ANOVA with Dunnett’s post hoc test for multiple comparisons. *n* = 6 controls, 7 Beta-Ac, 9 LPS, and 4 LPS + Beta-Ac.

**Figure 4:**
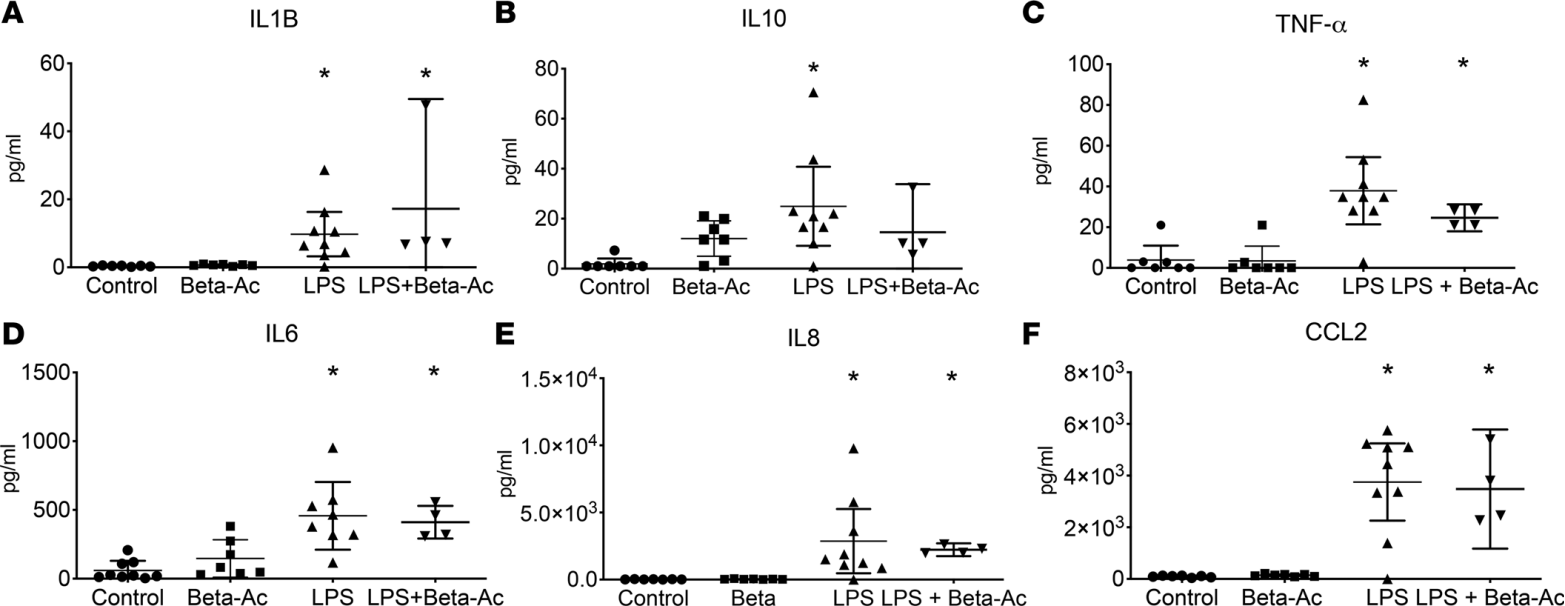
Combined intraamniotic LPS and Beta-Ac treatments given at the same time do not suppress cytokine-mediated inflammation of the bronchoalveolar lavage fluid at 5 days. (**A**–**F**) Concentrations of IL-1B (**A**), IL-10 (**B**), TNF-α (**C**), IL-6 (**D**), IL-8 (**E**), and CCL2 (**F**) were measured by ELISA in the bronchoalveolar lavage fluid. Intraamniotic LPS increased the concentrations of all cytokines measured in the amniotic fluid, but treatment with Beta-Ac did not reduce their concentrations at 5 days. Data presented as mean ± 95% CI with individual data points. *P < 0.05 by ANOVA with Dunnett’s post hoc test for multiple comparisons. *n* = 7 controls, 7 Beta-Ac, 9 LPS, and 4 LPS + Beta-Ac.

Given the antagonizing effect of corticosteroids on inflammatory response, we investigated whether the combined treatment reduced maternal-fetal inflammation by analyzing peripheral blood counts and cytokine concentrations in the amniotic fluid by ELISA. Exposure to intraamniotic LPS induced maternal leukopenia with decreased proportion of neutrophils in the peripheral blood, which was not suppressed by treatment with Beta-Ac. On the other hand, intraamniotic LPS increased the proportion of neutrophils in the fetal peripheral blood (Supplemental Table 2). Analysis of the amniotic fluid and fetal BALF showed increased cell counts 5 days after exposure to intraamniotic LPS, which persisted with cotreatment with Beta-Ac. Exposure to intraamniotic LPS increased the concentrations of IL-1B, IL-6, IL-8, IL-10, CCL2, and TNF-α in the amniotic fluid measured by ELISA, with similarly elevated levels in the combined LPS + Beta-Ac exposure (Figure 3). Similarly, cytokine analysis of the BALF of the rhesus macaque fetuses showed increased concentration of IL-1B, IL-6, IL-8, IL-10, CCL2, and TNF-α, with similar levels on the LPS + Beta-Ac exposure group (Figure 4). These results show that 5 days of exposure to intraamniotic LPS and the combined exposure of LPS + Beta-Ac induce a fetal inflammatory response. The concomitant treatment with Beta-Ac did not seem to suppress the cytokine expression at 5 days, although our interpretation is limited by the sample size in the combined exposure group.

### Intraamniotic LPS induces maturational-like signaling in the lung transcriptome.

**Figure 5:**
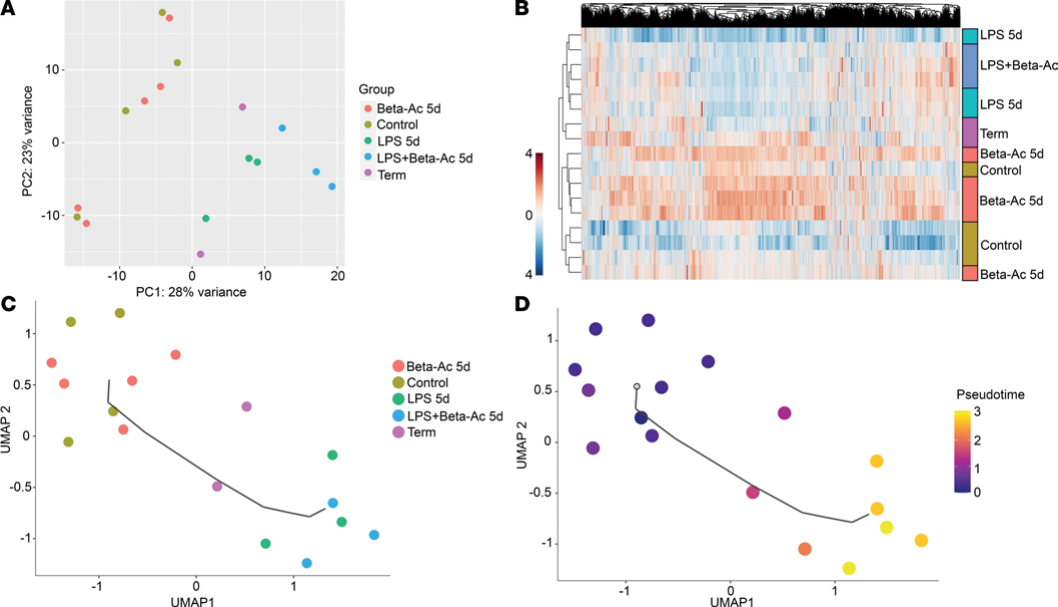
Lung transcriptome analysis of preterm rhesus macaque fetuses exposed to intraamniotic LPS demonstrate gene expression pattern similar to term animals. (**A** and **B**) Principal component analysis (**A**) and hierarchical clustering (**B**) of lung transcriptomes show clustering of preterm LPS exposed–animals with or without treatment with Beta-Ac, and those animals were more similar to term controls than to preterm controls. The effects of Beta-Ac treatment at 5 days had largely waned to a transcriptome pattern similar to preterm controls. (**C** and **D**) Reconstructed transcriptome trajectories on pseudotemporal analysis with Monocle 3 with the root on the preterm control cluster. Preterm controls, term controls, and intraamniotic LPS–exposed animals with and without Beta-Ac were aligned in a trajectory starting with preterm controls and ending with the intraamniotic LPS–exposed animals, suggesting greater differentiation on the latter. *n* = 4 preterm controls, 5 Beta-Ac 5d, 3 LPS 5d, 3 LPS + Beta-Ac, and 2 term controls.

We sought to determine the processes and mechanisms through which intraamniotic LPS induces fetal lung maturation and the mechanisms of maturation enhancement by treatment with ACS through RNA sequencing (RNA-Seq) analysis of the lungs from preterm rhesus macaques treated at 127 days of gestation and delivered after 5 days. We used our previously published dataset of preterm rhesus macaques exposed to Beta-Ac alone and delivered after 5 days for comparison (17). Principal component analysis and hierarchical clustering of expressed genes showed clustering of intraamniotic LPS, intraamniotic LPS + Beta-Ac, and term animals delivered at 157 days of gestation, suggesting that intraamniotic LPS induced transcriptional changes to the preterm lung similar to normal advancing gestational age (Figure 5, A and B). In contrast, the animals exposed to the Beta-Ac alone clustered with control animals, consistent with our previous observation of decreased transcriptomic changes by 5 days after treatment with Beta-Ac alone, with the physiological effects of increased lung compliance still present at this time point. We used pseudotemporal analysis on Monocle (19) to identify developmental trajectories among the LPS- and Beta-Ac–exposed animals relative to preterm and term controls. Group assignments are shown in Figure 5C, and the relative position of each animal along the pseudotemporal trajectory is shown in Figure 5D. Preterm controls, term controls, and intraamniotic LPS–exposed animals at 5 days were predicted to be in a continuum with the LPS-exposed animals farthest from the preterm controls and term animals between the preterm controls and LPS-exposed animals, indicating prenatal inflammation induced maturation-like changes. As the transcriptional effects of Beta-Ac alone wane by 5 days, these animals clustered — along with preterm controls — on pseudotemporal analysis.

### Intraamniotic LPS induces distal epithelial and suppresses proliferative mesenchymal progenitors in the preterm lung.

**Figure 6:**
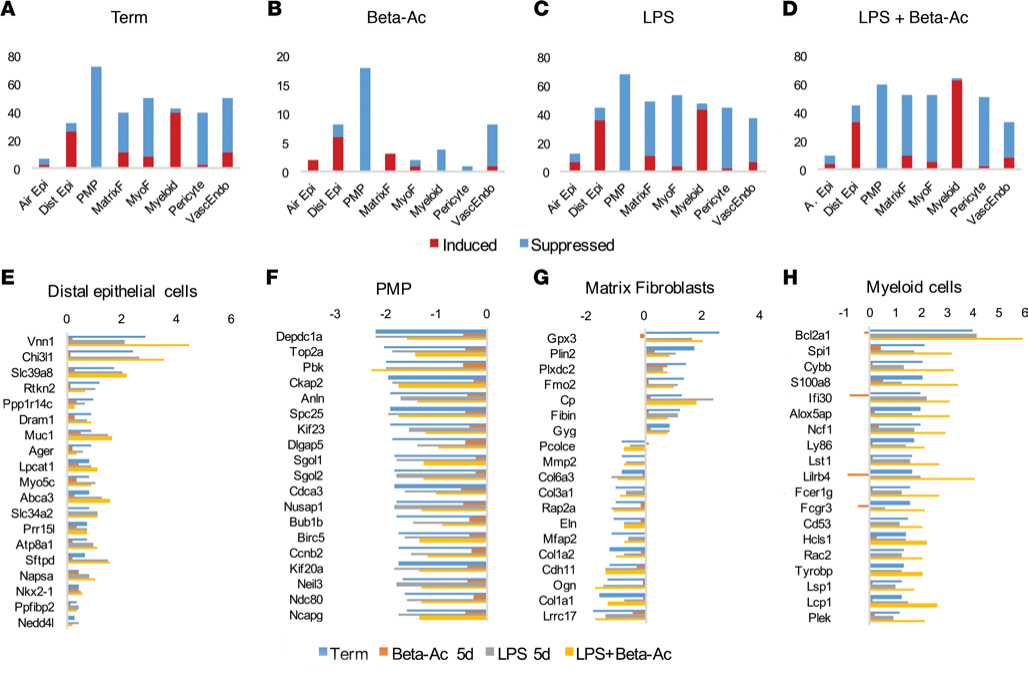
Prenatal inflammation induces mesenchymal and epithelial cell differentiation. Number of signature genes for different lung cell types differentially expressed by intraamniotic LPS and Beta-Ac. (**A**) Term controls versus preterm controls. (**B**) Beta-Ac 5 days versus preterm controls. (**C**) Intraamniotic LPS 5 days (5d) versus preterm controls. (**D**) Intraamniotic LPS + Beta-Ac 5d versus preterm controls. Exposure to intraamniotic LPS induced signature genes for matrix fibroblasts while suppressing signature genes for proliferative mesenchymal progenitors. Even the small number of genes that were differentially expressed by Beta-Ac at 5d showed suppression of proliferative mesenchymal progenitors and induction of distal epithelial cells and matrix fibroblasts signature genes. A similar pattern was seen after combined LPS + Beta-Ac. (**E**–**G**) Fold-change for top differentially expressed signature genes for distal epithelial cells (**E**), proliferative mesenchymal progenitors (**F**), matrix fibroblasts (**G**), and myeloid cells (**H**) are shown. Top differentially expressed signature genes for distal epithelial cells and proliferative mesenchymal progenitors were consistent between LPS, Beta-Ac, and the combined exposure. *n* = 4 preterm controls, 5 Beta-Ac 5d, 3 LPS 5d, and 3 LPS + Beta-Ac.

We determined the presence of cell type–specific signature genes using data from the single-cell RNA-Seq experiment of fetal lungs available on LungMAP (20, 21). Differentially expressed genes in term controls and animals exposed to Beta-Ac, LPS, or LPS + Beta-Ac relative to preterm controls were mapped to signature genes for the different cell populations identified by RNA-Seq in mouse lungs. Analysis of differentially expressed genes in term lungs compared with preterm controls showed that term lungs had suppression of signature genes for proliferative mesenchymal progenitors and induction of signature genes for distal epithelial cells and myeloid cells (Figure 6A). A similar pattern was observed with exposure to Beta-Ac alone, LPS, and LPS + Beta-Ac. The number of signature genes associated with the different cell populations in the lung was smaller after treatment with Beta-Ac compared with exposure to LPS or in term animals, likely reflecting the overall low number of differentially expressed genes with Beta-Ac after 5 days as discussed above (Figure 6, A–D). Exposure to intraamniotic LPS induced epithelial cell signature genes associated with surfactant transport (*ABCA3*, *LPCAT1*) and with immune response (*SFTPD*, *LAMP3*, *LPC2*). Interestingly, term gestation was associated with increased signature genes for myeloid cells with a similar number of induced genes as in LPS-exposed preterm animals. Most signature genes for matrix fibroblasts were suppressed by exposure to LPS or LPS + Beta-Ac with some induced genes. This pattern was similar to the pattern observed in term animals and the suppressed genes associated with matrix fibroblasts, which were largely extracellular matrix–associated genes such as *COL1A1*, *COL1A2*, and *ELN* (Figure 6, E–H). On the other hand, Beta-Ac treatment induced the only 3 matrix fibroblast signature genes that were differentially expressed by the treatment.

### Prenatal inflammation induces biological maturation.

**Figure 7:**
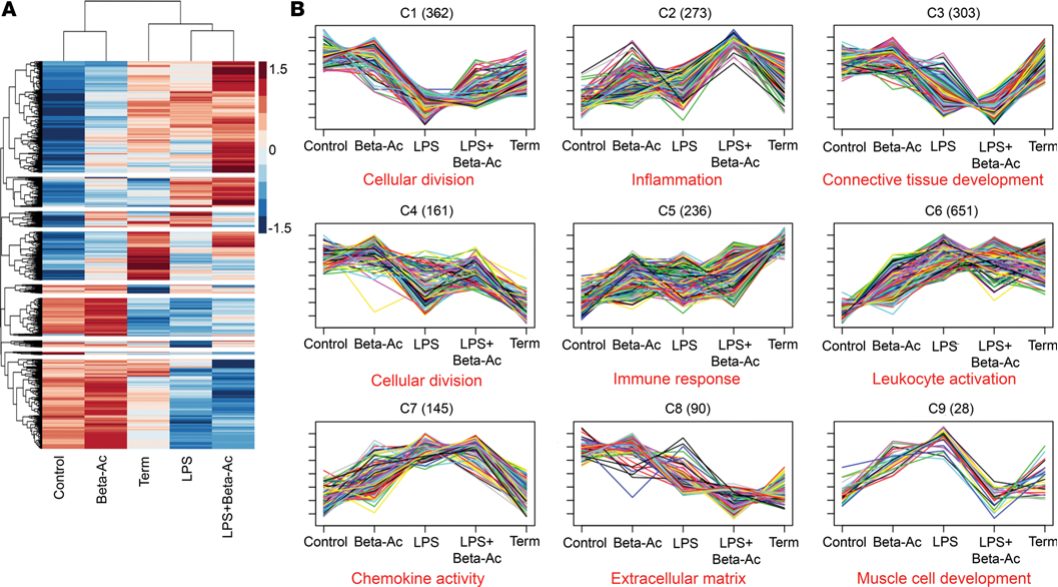
Differential expression and clustering analyses show the effects of intraamniotic LPS and its interaction with Beta-Ac on developmental processes. (**A**) Heatmap of differentially expressed genes showing relative expression. (**B**) Expression patterns identified in the 9 clusters with the number of genes displayed next to the cluster number. LPS 5d, LPS + Beta-Ac, and term animals had suppression of genes associated with connective tissue development and cellular division, as well as induction of genes associated with immune activation relative to preterm controls. *n* = 4 preterm controls, 3 LPS 5d, 4 LPS 16h, 3 LPS + Beta-Ac, and 2 term controls.

To identify similarities and dissimilarities on gene expression patterns among maturation induced by Beta-Ac, LPS, LPS + Beta-Ac, and normal lung maturation, we performed differential expression analysis and clustering of differentially expressed genes based on gene expression patterns. We also performed RNA-Seq of lung tissue from 2 near-term animals (155 days of gestation) to identify a mature lung transcriptome pattern (term group) (Figure 7). We identified 9 unique clusters and, with gene set enrichment analysis, identified the main biological process themes for each cluster based on the number of associated genes and adjusted *P* values (Figure 7B). Gene expression patterns show that LPS and LPS + Beta-Ac at 5 days had similar effects on major biological processes involved in lung maturation — in particular, the suppression of cellular division (clusters 1 and 4), suppression of connective tissue and extracellular matrix development (clusters 3 and 8), and activation of immune response (clusters 5 and 6). Treatment with LPS + Beta-Ac animals had a differential effect on these clusters, with a more profound suppression of genes associated with connective tissue and extracellular matrix (clusters 3 and 8) compared with LPS alone, more closely resembling the term expression pattern for genes in cluster 8, while suppressing genes that were not affected by normal maturation in cluster 3. On the other hand, LPS alone significantly suppressed genes in cluster 1, which was associated with cellular division. Interestingly, inflammation-related genes in cluster 2 were induced by Beta-Ac treatment and the combined LPS + Beta-Ac exposure, but not by LPS. Therefore, the combined exposure with LPS + Beta-Ac shared features with both Beta-Ac alone and LPS alone. These patterns demonstrate that treatment with ACS in inflammation-exposed animals promotes fetal lung maturation by regulating similar biological processes to normal fetal lung maturation but through some different genes and pathways involved in these processes.

### Prenatal inflammation and corticosteroids interact to amplify signaling for maturation of the lung extracellular matrix.

**Figure 8:**
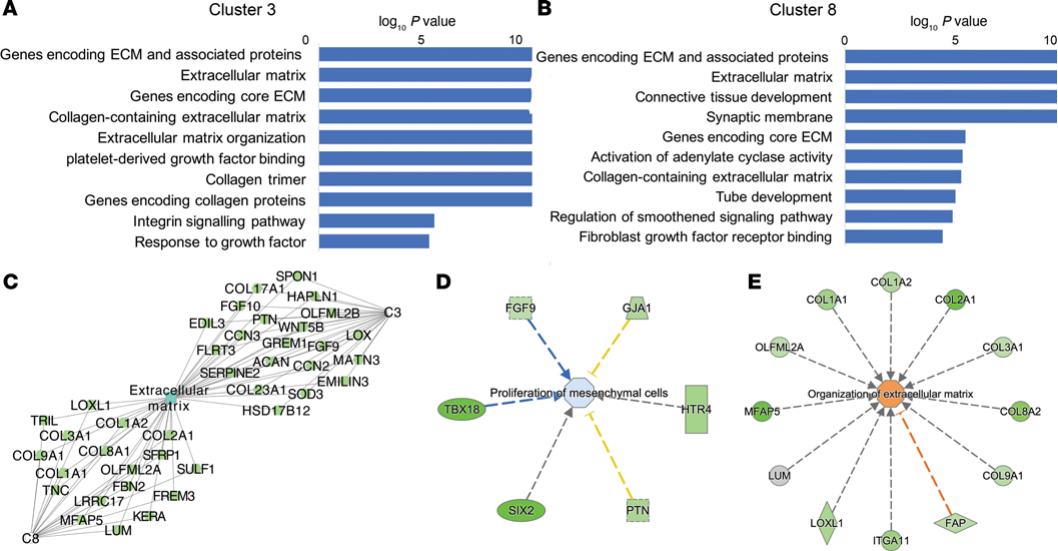
Intraamniotic LPS and Beta-Ac interact to augment suppression of mesenchyme development in the fetal lung. (**A**) Gene set enrichment analysis of genes in cluster 8 shows that genes in this cluster, which is suppressed in the lung 5d after LPS + Beta-Ac and in term lungs, are largely associated with extracellular matrix–related proteins and processes. (**B**) Gene set enrichment analysis of genes in cluster 3 shows that genes in this cluster, which is suppressed in the lung 5d after LPS + Beta-Ac but not in term lungs, are associated with extracellular matrix–related proteins and developmental pathways of the fetal lung, such as smoothened signaling and fibroblast growth factor. (**C**) Genes associated with extracellular matrix in clusters 3 and 8. (**D** and **E**) Ingenuity pathways analysis predicted suppression of proliferation of mesenchymal cells by genes in cluster 3 (**D**) and extracellular matrix by genes in cluster 8 (**E**). *n* = 4 preterm controls, 3 LPS 5d, 3 LPS + Beta-Ac, and 2 term controls.

A main characteristic of fetal lung maturation during transition toward the saccular and then alveolar stages is thinning of the mesenchyme, which improves lung compliance and brings the airspace and the capillaries in closer juxtaposition, facilitating gas exchange. The interaction of Beta-Ac and intraamniotic LPS induced further downregulation of genes associated with extracellular matrix compared with LPS or Beta-Ac alone, but similar to term controls (Figure 7B, cluster 8). These genes are associated with “extracellular matrix and associated proteins,” “collagen containing extracellular matrix,” and “integrin signaling” (Figure 8A). Moreover, the combination of Beta-Ac and intraamniotic LPS also suppressed genes in cluster 3, again beyond the exposure to LPS alone. Overall, genes in cluster 3 were not regulated by Beta-Ac treatment alone at 5 days. Genes in this cluster, however, were not differentially expressed in term lungs compared with preterm (Figure 7B, cluster 3). These genes are also associated with “extracellular matrix and associated proteins” and “collagen-containing extracellular matrix,” similar to cluster 3, as well as “connective tissue development,” “regulation of smoothened signaling pathway,” and “fibroblast growth factor receptor binding” (Figure 8B). Genes associated with “extracellular matrix” processes from both cluster shows that cluster 3 includes important lung development regulators such as *FGF9*, *FGF10*, and other *WNT* signaling–related molecules; cluster 8 includes several collagen proteins including *COL1A1* and *COL1A2*, which are components of type I collagen, the main collagen in lung (Figure 8C). Pathway analysis of genes in cluster 3 predicted that the suppression of *FGF9* and other genes in this cluster regulate the suppression of proliferation of mesenchymal cells, consistent with the suppression of signature genes for proliferative mesenchymal progenitors (Figure 8D). Genes in cluster 8, including *COL1A1* and *COL1A2*, were predicted to regulate organization of the extracellular matrix (Figure 8E).

### Intraamniotic LPS induces cellular death of lung connective tissue.

**Figure 9:**
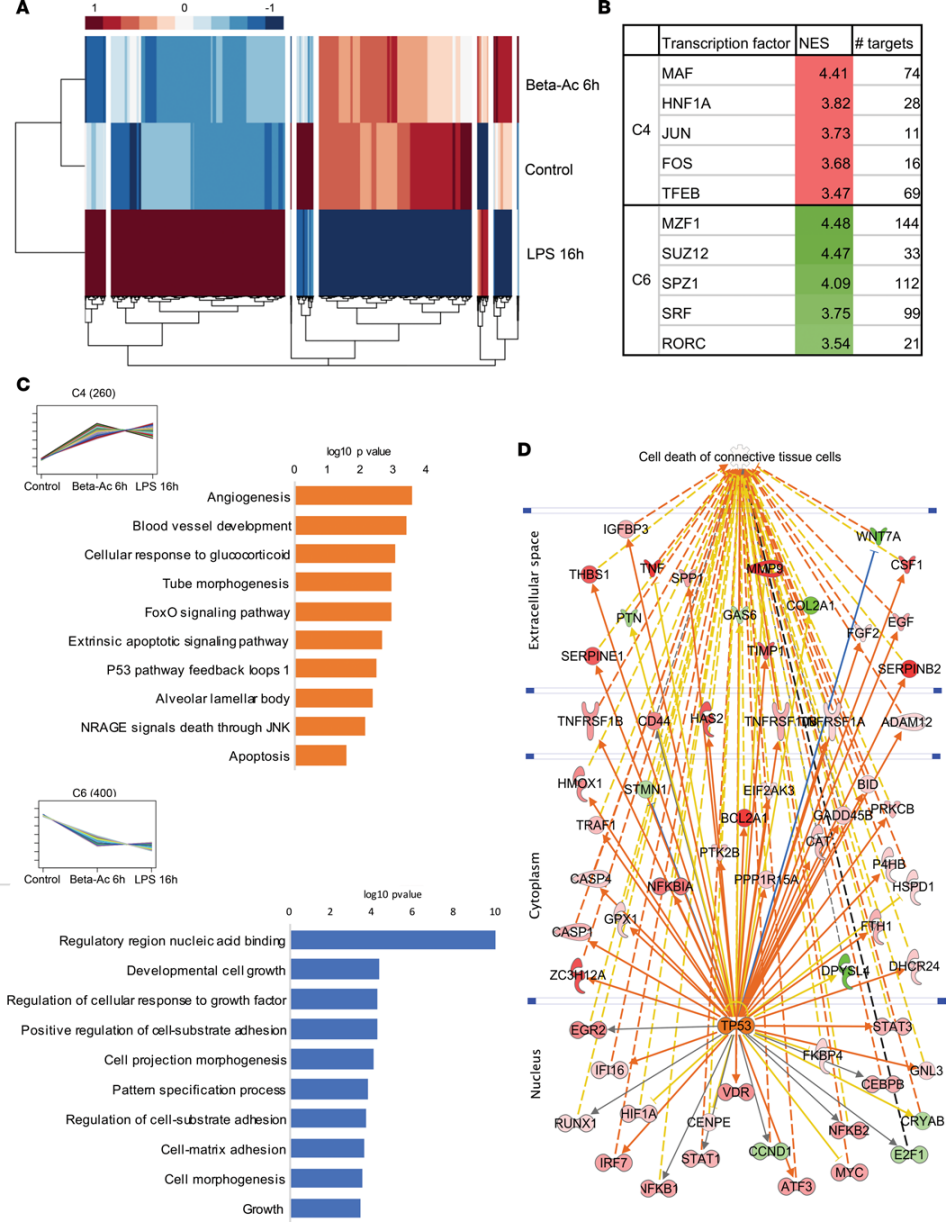
Intraamniotic LPS and Beta-Ac induce cellular apoptosis of connective tissue cells via Tp53 signaling. (**A**) Heatmap of differentially expressed genes in preterm rhesus macaque fetuses exposed to intraamniotic LPS 16h prior to delivery or to maternal intramuscular Beta-Ac 6h prior to delivery at 130 days of gestation (80% gestation). On differential expression and cluster analysis based on gene expression patterns, we identified 8 unique clusters. (**B**) Transcription factor prediction analysis showing transcription factors associated with Cluster 4 (C4) of genes induced by intraamniotic LPS and Beta-Ac and with cluster (C6) of genes suppressed by intraamniotic LPS and Beta-Ac. (**C**) Gene set enrichment analysis of genes in C4 showed association of genes in this cluster with “angiogenesis,” “apoptosis,” and “p53 pathway” and analysis of genes in C6 showed associated with “developmental cell growth,” “cell projection morphogenesis,” and “cell matrix adhesion.” (**D**) Network analysis of genes differentially expressed by intraamniotic LPS at 16h predicted upregulation of p53 signaling, which was associated with predicted induction of apoptosis of connective tissue cells through several differentially regulated proteins. *n* = 4 preterm controls, 3 Beta-Ac 6h, 4 LPS 16h.

To understand the early signaling mechanisms that drive the structural lung maturation, we exposed another group of pregnant rhesus macaques with intraamniotic LPS at 130 days of gestation and delivered them 16 hours later, in comparison to the early transcriptome effects of Beta-Ac on the fetal lung, which we have previously published (17). In brief, in the previous study, we treated rhesus macaque fetuses with Beta-Ac (0.125 mg/kg) and delivered them by cesarean section delivery 6 hours after treatment, which is the time of peak betamethasone concentration in the fetal plasma after maternal intramuscular injection (22). Differential expression analysis of transcriptome data and clustering for differentially expressed genes revealed, in 2 clusters of genes, that Beta-Ac (6 hours) and intraamniotic LPS (16 hours) had similar expression effects (Figure 9A). Cluster 4 was composed of genes induced by both LPS and Beta-Ac, which were associated with “angiogenesis,” “blood vessel development,” “tube morphogenesis,” “apoptosis,” and “p53 pathway” (Figure 9C, top). Transcription factor binding site analysis was performed using iRegulon based on ENCODE data (23, 24). The top transcription factors predicted to regulate the induced genes in cluster 4 were MAF, HNF1A, JUN, FOS, and TFEB, ordered by their normalized enrichment score (NES) (Figure 9B). Cluster 6 was composed of genes suppressed by both Beta-Ac and LPS and were associated with “developmental cell growth,” “cell projection morphogenesis,” “regulation of cell-substrate adhesion,” and “growth” (Figure 9C, bottom). The top transcription factors predicted to regulate the suppressed genes in cluster 6 were MZF1, SUZ12, SPZ1, SRF, and RORC. Both intraamniotic LPS and ACS induced apoptosis-related genes and suppressed developmental pathways. Network analysis of differentially expressed genes predicted activation of TP53 pathways with associated induction of cell death of connective tissue cells through induced and suppressed downstream molecules (Figure 9D), suggesting that suppression of the extracellular matrix at 5 days was associated with early signaling of mesenchymal cells.

## Discussion

More than 85% of infants delivered preterm are exposed to prenatal inflammation (10), and in high–medical resource settings, more than 90% of preterm infants receive ACS prior to preterm delivery. ACS has multiple effects on the fetal lung, including thinning of the mesenchyme, improved water tissue balance, and increased surfactant production, which results in improved lung compliance, decreased respiratory distress syndrome, and decreased mortality. Improvements in lung compliance result in decreased need for respiratory support and decreased lung injury from mechanical ventilation and oxygen exposure. The effects of LPS and interaction with ACS are less well understood. Understanding how these exposures interact to modulate the preterm lung development and maturation is critical if we are to improve the respiratory management and outcomes of these infants. We report, in a preterm nonhuman primate model, that prenatal inflammation improves lung compliance and that treatment with ACS has an additive effect to augment fetal lung maturation more than either exposure alone. These findings suggest an early benefit of improved respiratory function among preterm infants treated with ACS in the setting of mild to moderate prenatal inflammation. However, this benefit may come at a long-term cost of increased BPD, as suggested by studies demonstrating the association between chorioamnionitis and BPD (25, 26), because the maturation is artificially induced and not natural as gestational age progresses.

While inflammation-induced lung maturation and its interaction with ACS has been previously reported in preterm sheep (8, 13), there are 2 clinically important differences in the present study. First, we used a corticosteroid dose that is 25% lower than the one used in sheep studies in the form of Beta-Ac alone, which causes much lower maternal and fetal peak plasma levels of betamethasone but still causes fetal lung maturation (17). The enhanced fetal lung maturation we observed with LPS + low-dose Beta-Ac was comparable with the enhancement observed in fetal sheep exposed to the standard clinical dose and LPS (13). Second, we report this interaction in nonhuman primates, strongly suggesting that this interaction also happens in humans. Moreover, while the role of glucocorticoid signaling and ACS treatment on developmental processes has been more extensively evaluated in KO animals (27–29) and in translational models using nonhuman primates (3, 30), the short- and long-term effects of inflammation on lung development is less known.

Thinning of the mesenchyme by glucocorticoid during normal fetal lung maturation has been attributed mostly to suppression of proliferation seen in KO mice for the glucocorticoid receptor (31, 32). Transcriptome analysis shows that both treatment with exogenous glucocorticoid as Beta-Ac and exposure to intraamniotic LPS cause an activation of cellular death and senescence pathways via activation of p53 signaling early after exposure. The combination of these exposures led to suppression of signature genes for proliferative mesenchymal cells and induction of signature genes for distal alveolar epithelial cells after 5 days. While suppression of cellular proliferation is known to be necessary for normal fetal lung maturation, cell death also has an integral role in these processes. During embryonic and fetal lung development in rodents, inhibition of autophagy decreases branching morphogenesis, inhibits thinning of the mesenchyme, and decreases saccular development, leading to neonatal death from respiratory insufficiency (33). Later, during the alveolar phase of lung development, there is an increase in programmed cell death of mostly fibroblasts and type 2 alveolar cells, which peak at 3 weeks of life in rodents (34). The physiologic phenotype of this cell loss in the mesenchyme is an increase in potential lung gas volume prior to an increase in surfactant at 1 or 2 days in the sheep (35). ACS induce mesenchyme thinning and surfactant production by poorly understood mechanisms. In ACS-induced maturation, mesenchymal thinning precedes increases in pulmonary surfactant, with some improvement in lung compliance prior to increase in surfactant production (30, 36). While surfactant protein mRNA changes happen as early as 4–6 hours, changes in surfactant content can only be detected by 2–3 days; the effects on surfactant protein mRNA is reversed after about 7 days, followed by loss of the benefit in RDS reduction that is observed clinically 7–10 days after treatment (3, 37). While the risk of RDS is dependent on sex — with preterm males more likely to develop RDS than females — we did not observe sex-specific differences in lung compliance or response to Beta-Ac or LPS, although our sample size was too limited for this comparison.

We found that, 16 hours after exposure to intraamniotic LPS, there is an upregulation of programmed cell death and cellular senescence pathways mediated via TP53. Among the predicted transcription factors to regulate genes induced by inflammation and corticosteroids is JUN, with 11 targets. JUN expression progressively increases in the mesenchymal-derived cell during normal lung development in mice from E16.5 to P28 (20, 21), suggesting an important role in mesenchymal maturation during normal lung development and a potential mechanism through which inflammation and ACS induce fetal lung maturation. ChIP-Seq experiments show glucocorticoid receptor (NR3C1) binding in the regulatory region of Jun (24, 38). Moreover, inflammation is a well-established driver of Jun activation through increase in cytokines and binding of TLRs including TLR4, which binds LPS (39). Thus, Jun could represent a common pathway in inflammation- and corticosteroid-induced fetal lung maturation. The TLR4 signaling pathway also regulates nitric oxide production in the pulmonary endothelial cells, which participates in epithelial cell maturation into type 2 alveolar cells and production of surfactant proteins C and B in these cells (40), demonstrating a direct role of innate immunity on fetal lung maturation.

The early activation of cell death was associated with a suppression of extracellular matrix development–related genes and processes. Our observation of suppression of signature genes associated with matrix fibroblasts in LPS-exposed animals may be due to the induction of mesenchymal cell death in association to maturation, leading to an overall reduction in mature fibroblasts. A similar pattern was also observed in term animals, with some matrix fibroblast signature genes being induced but most being suppressed. Thinning of the mesenchyme has been described in the sheep model of fetal lung maturation induced by corticosteroids and by intraamniotic LPS (35, 41). We observed suppression of the 2 main lung collage chains *COL1A1* and *COL1A2* after intraamniotic LPS + Beta-Ac. Suppression of collagen and other extracellular matrix genes represent a large proportion of suppressed signature genes for matrix fibroblasts after LPS exposure. While the total lung collagen content increases with lung growth and maturation, the synthesis rate of collagen decreases from 80% gestation to full term (35, 42). Collagen is responsible for the lung structure, but it does not contribute to the lung compliance and elasticity, which is provided by the elastic fibers — mainly elastin and microfibrils (43). Single-cell RNA-Seq of rodent lungs show progressive increase in the mRNA for elastin (*ELN*), *COL1A1*, and *COL1A2* in mesenchymal cell differentiation (20). Intraamniotic LPS, however, suppressed ELN as previously observed in a preterm lamb model (44). The combined treatment with Beta-Ac did not antagonize the decrease in *ELN* caused by intraamniotic LPS. Suppression of elastin may result in an abnormal saccular and alveolar lung development and may contribute to the higher risk of BPD seen in infants exposed to prenatal inflammation (25, 45) and increased tendency of steroid-exposed fetal lungs to rupture with mechanical ventilation (46).

Glucocorticoid signaling in the fetal lung mesenchyme is critical for fetal lung maturation from the pseudoglandular into the canalicular and cellular stages. Deletion of the glucocorticoid receptor in mesenchymal cells results in arrest of lung maturation in the pseudoglandular stage and early neonatal mortality, while deletion of the glucocorticoid receptor restricted to epithelial cells causes delayed maturation with no effect on neonatal survival (29). Meanwhile, both the combination of intraamniotic LPS + Beta-Ac and term animals showed suppression of genes related to developmental pathways of lung development relative to control, including *FGF9* and *FGF10*. Both fibroblast growth factors are essential for lung development, and KO of either *FGF9* or *FGF10* causes early neonatal mortality due to lung hypoplasia (47, 48). FGF9 promotes mesenchymal and epithelial cell proliferation (49); therefore, its suppression is consistent with a more mature and differentiated lung at term gestational age and with the maturational effect of the combination of intraamniotic LPS + Beta-Ac. FGF10 is a mesenchymal-derived growth factor that controls lineage commitment and proliferation of alveolar epithelial cells (50). While decreased by intraamniotic LPS + Beta-Ac at 5 days, *FGF10* is induced 16 hours after exposure to intraamniotic LPS and is likely to play a key role in alveolar epithelial cell differentiation and increased surfactant production induced by inflammation and ACS. The late suppression of these genes in the LPS + Beta-Ac may result from a more mature lung structure than either single treatment alone, as observed in the improved lung compliance and higher satPC concentrations.

One of the limitations of our findings is that human chorioamnionitis is an ongoing subacute-to-chronic inflammatory exposure caused by ongoing infection of the chorioamnion and amniotic compartment. While our model does not recapitulate ongoing inflammatory stimuli (instead, a single exposure to LPS), the cytokine levels in the amniotic fluid observed at 5 days after intraamniotic (IA) LPS is similar to the previously reported levels at 16 hours after IA LPS (16). Also, we used the minimally effective dose of Beta-Ac to induce fetal lung maturation that we previously identified (3). The standard clinical dosing includes betamethasone-phosphate, which results in high maternal and fetal peak levels and is given as 2 doses 24 hours apart, which prolongs the maternal and fetal exposure. The overall lower exposure to corticosteroids with the low-dose Beta-Ac compared with the standard clinical dose may result in differential gene expression regulation, even with similar enhancement of fetal lung maturation.

In conclusion, we defined the fetal lung response to prenatal inflammation and the interaction with ACS in a nonhuman primate model. The improved lung mechanical function observed suggests that preterm infants exposed to prenatal inflammation may have an additional benefit of improved lung mechanics and surfactant concentration, even when exposed to doses of corticosteroids much lower than those clinically used. This may be particularly relevant for low-resource settings where the clinical dose of ACS caused increased neonatal mortality, associated with increased perinatal infection (14, 51). In these settings, a lower dose of ACS may be beneficial to reduce the risks associated with therapy while maintaining the benefits of enhanced lung maturation.

## Methods

### Animals.

Time-mated pregnant rhesus macaques at 127 days of gestation were treated with either intraamniotic LPS 1mg (*E*. *coli* O55:B5, Sigma-Aldrich) diluted in 1 mL of sterile saline (LPS), intramuscular Beta-Ac (0.125 mg/kg; a gift from Merck Sharp & Dohme), or intraamniotic LPS + Beta-Ac. Previous studies from our group comparing Beta-Ac dosing with the clinical treatment consisting of the 1:1 mixture of Beta-Ac + betamethasone-phosphate have identified that 0.125 mg/kg of Beta-Ac is the minimally effective dosing to induce fetal lung maturation in preterm rhesus macaques and in sheep fetuses (3, 52). To determine the developmental pathways modulated by LPS, animals were delivered at 16 hours or 5 days after intraamniotic LPS. To determine the maturational interaction between intraamniotic LPS and steroids, animals that received Beta-Ac or LPS + Beta-Ac were delivered 5 days after treatment at 132 days of gestation. Preterm controls were delivered at 132 days of gestation, and term controls were delivered at 155 days of gestation. Preterm and term controls did not receive any intervention. No spontaneous labor or fetal losses were observed in the intervention or control groups.

At delivery, we collected amniotic fluid for measurement of protein cytokines by ELISA. We measured pressure-volume curves by inflating lungs to 40 cmH_2_O, followed by deflation with measurements of lung volumes using a syringe and pressure manometer. BALF was recovered from the right lung; the right upper lobe of the fetal lung was inflation fixed with formalin at 30 cm H_2_O pressure for histology, and the left lung was snap frozen for RNA-Seq.

### Immunofluorescence and confocal microscopy.

Paraffin-embedded tissue sections underwent heat-assisted antigen retrieval with citrate buffer (pH 6.0), followed by blocking with donkey or goat serum and incubation with primary antibodies overnight (Supplemental Table 3): Nkx2.1 (Seven Hills Bioreagents, WRAB-1231), Nkx2.1 (in-house antibody, G237), pro-surfactant protein C (Seven Hills Bioreagents, WRAB-9337), ABCA3 (in-house antibody, GP985), and smooth muscle actin (Santa Cruz Biotechnology, A5228). After that, sections were incubated with Alexa Fluor antibody against the host species of the primary antibody (Invitrogen), followed by DAPI (Invitrogen, dilution 1:2000). Sections were mounted with ProLong Gold (Invitrogen). Stained slides were imaged by confocal microscopy for colocalization of fluorescent antibodies at 20× magnification, 1024 × 1024 pixels resolution, on a Nikon Eclipse A1RSi inverted microscope (Nikon Instruments Inc.).

### SatPC isolation and measurement.

Lipids were extracted from the BALF with chloroform-methanol (2:1). SatPC was isolated after exposure to osmium tetroxide and quantified by phosphorus assay as previously described (53).

### RNA isolation and sequencing.

Total RNA was extracted from frozen lung tissues using the RNeasy Universal Mini Kit (Qiagen) according to the manufacturer’s instructions. RNA quality and integrity was verified using the Agilent 2100 Bioanalyzer (Agilent Technologies). All samples had RNA integrity number > 8. RNA-Seq was performed by the Cincinnati Children’s Hospital Medical Center DNA Sequencing and Genotyping Core with a read depth of 20–30 million reads per sample for 75 bp paired-end reads. The raw sequence reads in FASTQ format were aligned to the Rhesus (*Macaca mulatta*) genome build MMUL10 using kallisto (54) followed by gene summarization with tximport (55). After checking data quality, differential expression analyses comparing treatment groups with control and between each other were performed using DESeq2 with false discovery adjustment (56). Genes were considered differentially expressed based on their fold change relative to control (≥2), *P* value (<0.05), and *q* value (<0.05).

### Functional enrichment and pathway analysis.

Lists of differentially expressed genes were used for functional enrichment analysis of Gene Ontology (GO) and pathway terms using the ToppCluster web server (57). Only unique terms associated with either induced or suppressed genes and at least 2 genes are reported. Negative log *P* values represent terms associated with suppressed genes, and positive log *P* values are associated with induced genes.

### Cytokine concentration measurement.

We measured cytokine concentration in the amniotic fluid using Luminex technology using multiplex kits for nonhuman primate (MilliporeSigma). Concentrations were calculated from standard curves for recombinant proteins.

### Availability of data and materials.

The gene expression data have been deposited in NCBI’s Gene Expression Omnibus (GEO) and are accessible through GEO Series accession no. GSE148645 (https://www.ncbi.nlm.nih.gov/geo/query/acc.cgi?acc= GSE148645). The gene expression data for Beta-Ac–treated animals have been previously published (3) and are accessible through GEO Series accession no. GSE118438 (https://www.ncbi.nlm.nih.gov/geo/query/acc.cgi?acc= GSE118438).

### Statistics.

We used GraphPad Prism 8 for statistical analyses (GraphPad Software Inc.). Continuous variables were analyzed using 1-way ANOVA with Tukey’s post hoc for multiple testing or Krukal-Wallis as appropriate. Differences were considered significant for *P* < 0.05.

### Study approval.

The University of California Davis IACUC approved all animal procedures, which were performed at the California National Primate Research Center in Davis, California, USA.

## Author contributions

AFS, CAC, LAM, PP, SGK, and AHJ initiated and designed the study. AFS, PSK, PP, JB, and CMJ acquired and processed the samples and generated the experimental data. SGK, CAC, and AHJ supervised the research. AFS wrote the manuscript. All authors contributed to the writing and editing of the manuscript.

## Supplementary Material

Supplemental data

## Version 1 12/17/2020

Electronic publication

## Version 2 01/20/2021

Fixed spelling error
