# Supplementary material for: Prenatal inflammation enhances antenatal corticosteroid–induced fetal lung maturation
Source: JCI Insight. 2020 Dec 17;5(24):e139452. doi: 10.1172/jci.insight.139452 (PMC7819743; doi:10.1172/jci.insight.139452)
Supplement: Supplemental data [file jciinsight-5-139452-s160.pdf]

Supplementary table 1. Animal data and sample sizes

	Preterm	Beta-Ac		LPS		LPS+	Term
	control	6h	5d	16h	5d	Beta	control
n	7	3	7	4	9	5	2
n for sequencing	4	3	5	4	3	3	2
Gestational age at delivery (days)	133 ± 2.4	133 ± 0.5	131 ± 1.9	131 ± 0.4	132 ± 1.1	131 ± 0.4	157 ± 0.5
Fetal weight (g)	331 ± 34	331 ± 9.9	338 ± 39	338 ± 45	327 ± 35	298 ± 40	514 ± 40
Sex (M/F)	6/2	2/1	3/4	2/2	5/4	1/4	1/1

Supplementary table 2. Organ weights, alveolar wash cell count, amniotic fluid cell count, leukocyte counts and differentials. *p<0.05

	Preterm control	LPS 5d	LPS + Beta-Ac 5d
Lung weight (g)	7.7 ± 1.0	9.2 ± 1.6*	8.7 ± 1.3
Lung:Body weight ratio	23.5 ± 3.2	28.4 ± 2.8*	28.1 ± 3.4
Thymus weight (g)	1.1 ± 0.3	1.0 ± 0.2	1.0 ± 0.3
Thymus:Body weight ratio	3.3 ± 0.8	3.0 ± 0.5	3.1 ± 0.5
Cell count – alveolar wash ($x10^4$ /mL)	3 ± 1.2	39 ± 14*	41 ± 14*
Cell count – amniotic fluid ($x10^4$ /mL)	2 ± 2	28 ± 8*	37 ± 7*
Maternal WBC ($x10^9$ /L)	8.2	5.6*	8.5
Maternal neutrophils (%)	78 ± 6	51 ± 16*	62 ± 8
Fetal WBC ($x10^9$ /L)	2.4 ± 0.6	3.3 ± 0.8	4.2 ± 1.2*
Fetal neutrophils (%)	9.7 ± 6.1	23.3 ± 4.7*	22.8 ± 4.3*

Supplementary table 3. Primary antibodies used for immunofluorescence staining.

Antibody	Species	Catalog #	Company	Dilution
NKX2.1[1]	Rabbit	WRAB-1231	Seven Hills Bioreagents	1:500
NKX2.1[2]	Guinea pig	G237	In house	1:200
Pro-surfactant protein C (SFTPC)[1]	Rabbit	WRAB-9337	Seven Hills Bioreagents	1:100
ABCA3[2]	Guinea pig	GP985	In house	1:100
Smooth muscle actin (SMA)[3]	Mouse	A5228	Santa Cruz Biotechnologies	1:2000
